# Out of Sight, but Not Out of Mind: Aspects of the Avian Oncogenic Herpesvirus, Marek’s Disease Virus

**DOI:** 10.3390/ani10081319

**Published:** 2020-07-30

**Authors:** Irit Davidson

**Affiliations:** Division of Avian Diseases, Kimron Veterinary Institute, Bet Dagan 50250, Israel; davidsonirit@gmail.com

**Keywords:** poultry, chickens, tumors, Marek’s disease virus, feathers, immunodominant proteins, viral-induced tumors

## Abstract

**Simple Summary:**

Often tumors are eventually observed at poultry slaughter, leading to condemnation, not of zoonotic, but because of esthetic reasons. Most avian tumors are caused by infection with oncogenic viruses, where the most prominent is a herpesvirus, Marek’s disease virus (MDV). MDV-infections are economically important in the poultry industry as they cause immunosuppression, decreases in productivity and profitability and increased morbidity, mortality, and condemnation at slaughter. MDV was discovered about 50 years ago, and still its study is ongoing until now. This review is presenting the MDV biology, and protection by vaccination, but also topics that were less brought to wider knowledge, like the vaccine and virulent virus presence in feathers, and their use in assessing the efficacy of vaccines application in commercial settings. In addition, two relatively novel topics are reviewed, like the meq MDV oncogene and its recent implication in molecular epidemiology and MDV virulence determination, and second, the importance of native, three-dimensional conformational epitopes of the immunodominant glycoprotein B and the molecular recombination between DNA-DNA and DNA-RNA viruses. Our studies were particular, as they were originally describing how the native virion is activating the chicken immune response to create virus-neutralizing antibodies. The topic might shed light on items to be focused on in future searches for effective vaccines.

**Abstract:**

Marek’s disease virus is an economically important avian herpesvirus that causes tumors and immunosuppression in chickens and turkeys. The virus, disease, and vaccines have been known for more than 50 years, but as knowledge gaps still exists, intensive research is still ongoing. The understanding of MDV complexity can provide scientific insight in topics that cannot be experimented in humans, providing a unique model that is dually useful for the benefit of the poultry industry and for studying general herpesvirology. The present review presents the following topics: the MDV biology, the vaccine’s and virulent virus’ peculiar presence in feathers, protection by vaccination. In addition, two relatively behind the scenes topics are reviewed; first, the meq MDV oncogene and its recent implication in molecular epidemiology and in the MDV virulence determination, and second, the functionality of conformational epitopes of the MDV immunodominant protein, glycoprotein B. Our studies were particular, as they were the only ones describing three-dimensional MDV gB oligomers. MDV gB (glycoprotein B) continuous and discontinuous epitopes were shown to possess distinctive neutralization activities. In contrast, the significance of oligomerization of the viral membrane proteins for the creation of discontinuous epitopes in other herpesviruses was explored extensively.

## 1. The Biology of the Oncogenic Marek’s Disease Virus

Viral induced tumors in poultry are economically important for the poultry industry because they cause decreased productivity and profitability, due to increased morbidity and mortality of birds during growth, and increased condemnation at slaughter. In the study of avian tumors, attention has been focused on those of viral etiology. Studying avian tumorigenesis is important not only from the standpoint of its economic importance, but also as a potential model for tumorigenesis in humans [1,2]. The avian tumor viruses are not zoonotic and do not have any public health significance, however, poultry condemnation at slaughter is practiced because of esthetic reasons.

The avian oncogenic viruses include the herpes virus, Marek’s disease virus, and four retroviruses. The herpes virus is the DNA double stranded Marek’s Disease Virus (MDV) that causes tumors in chickens and turkeys [3,4,5,6,7]. MDV transforms T-lymphocytes, leading to the formation of skin and visceral tumors, but also causes immunosuppression (IS) and a variety of symptoms until tumors appear. In most cases the enlargement of the peripheral nerves, thickened proventriculus wall and breast muscle emaciation are evident and associated with the appearance of visceral tumors. The MDV-associated symptoms also include slow weight gain, uneven growth, enhanced mortality and morbidity, neurological and eye lesions. MDV is widely disseminated in poultry worldwide, therefore is considered ubiquitous. Natural MDV isolates of variable virulence have been isolated with pathogenicities classified as very virulent plus, very virulent, virulent, mild for serotype 1 MDVs, and avirulent strains for MDV serotypes 1, 2, and 3 [3], that have been denoted as *Gallid alphaherpesvirus* 2 (GaHV-2, referred to as MDV in this review), the avirulent *Gallid alphaherpesvirus* 3 (GaHV-3 or MDV-2) and *Meleagrid alphaherpesvirus* 1 (MeHV-1 or herpesvirus of turkey (HVT)), respectively, according to the latest ICTV nomenclature.

The IS in commercial poultry is economically important because of decreased productivity, increased mortality, fruitless investments in veterinary medical assistance and treatment, as well as increased susceptibility to infection by various pathogens and decreased efficacies of various vaccines [8]. The MDV-induced IS that is a rather complex condition, including two phases, the early IS phase, associated with early cytolytic infection of the lymphoid organs and the late IS phase, associated with the reactivation of MDV and development of tumors. The late phase is associated with the development of tumors and with deregulation of the immune responses during reactivation. During the early phase two processes occur: destruction of T and B lymphocytes due to the initial virus replication in the lymphoid organs and appearance of suppressor macrophages that inhibit the lymphocyte replication. Additional effects also occur, like down-regulation of class 1 and 2 major histocompatibility complex antigens, cytotoxic T lymphocytes impairment, lowering CD8 expression in peripheral T cells, induction of apoptosis in CD4+ T cells, a long-lasting B cell lymphopenia, and production of high levels of nitric oxide [9,10,11].

## 2. Feathers as the Main Site of Marek’s Disease Virus (MDV) Replication and Spread

MDV was the first and most extensively studied virus regarding its presence in feathers [12,13,14,15]. The virus replicates in the feather follicle epithelium (FFE), as the only anatomical site where MDV productive replication occurs and from where it spreads horizontally in poultry houses with dust and dander [16]. The infectivity of the cell free virus in feather dust can be blocked by commercial air filters and by keeping a strict hygiene of the poultry houses [16]. As MDV replicates in the feather follicle epithelium cells and spreads horizontally in the poultry houses, avoiding the environmental spread in commercial flocks is an important control measure. The horizontal spread is very efficient as it is made possible through infection of stratified squamous epithelium cells in the skin that commonly detach with molted feathers or during skin renewal, and via dust and dander, making the virus ubiquitous [16,17].

The feature of MDV replication in feather follicle epithelium motivated numerous studies that utilized the feathers to detect and isolate the virus. For first time we demonstrated the kinetics of MDV shedding by molecular amplification [18], later being confirmed by Baigent et al. [19]. We used the feathers as a supporting organ in the molecular diagnosis of commercial flocks [12]. We initially separated whole viruses from feather tip extracts by Pulsed Field Gel Electrophoresis [20], and as the basis for the usage of feather tip extracts for the developing an experimental infection model of MDV via mucosal surfaces of the eyes and airways of SPF chicks [21]. Moreover, by using the feather tips we developed originally [22], the quantitation of the corticosterone content, reflecting the viral-induced stress in experimentally MDV-infected SPF chicks.

MDV was the only poultry live vaccine whose vaccination uptake efficacy was studied by demonstrating its presence in feather tips. The presence of live vaccines was demonstrated in the feathers of experimentally infected and commercially vaccinated layers in Australia [23,24,25]. By developing a novel nested real-time PCR assay we were able to characterize the vaccine application efficacy in commercially vaccinated chicks in Israel [13] and to trace back MDV-affected chicken flocks to problematic MDV vaccine application [14].

## 3. Control by Vaccination

The worldwide intensification process of the poultry production, which occurred in the last 50 years, led to a dramatic increase in the incidence of MDV infections, where the tumors and mortality became a very costly component for the industry. As a result, researchers looked for effective vaccines that would protect poultry from mortality and could avoid condemnation at slaughter due to tumors. Avirulent viruses have been adapted to serve as effective vaccines against virulent MDVs, and prevention of disease. In that regard, the MD comprises a unique example in nature, where a malignant disease that is caused by a herpesvirus, can be effectively controlled by vaccination using naturally isolated avirulent MDVs.

The first MDV vaccine, which was developed by Kawamura et al. [26] and Witter et al. [27] in the 70’s, was based on the herpesvirus of turkeys (HVT) MDV serotype 3 (MDV-3). HVT was protective against MDV challenge of low virulence that prevailed in the [28]. The “dry” HVT vaccine (MDV-3 vaccine) was produced as the only cell-free MDV vaccine produced by sonication and lyophilization of infected cell cultures and is very well-known and used extensively because of the convenience of storage and handling. However, due to its susceptibility to neutralization by maternal antibodies the “dry” HVT vaccine was effective only against virulent MDV strains, but not against very virulent MDV isolates (vvMDV) that emerged beginning in the 80’s [29,30]. MDV vaccines based on the three serotypes (MDV-1, MDV-2, and MDV-3) were further developed as cell-associated viruses within living chicken embryo fibroblast cell cultures. To keep the MDV vaccine efficacy, the vaccine virus has to reside inside living cells. For that reason, the cell-associated vaccines require special handling and storage in liquid nitrogen until use. The maintenance of an appropriate cold-chain and fast and precise vaccine dilution and application of the MDV vaccine is demanding and crucial for acquiring proper protection.

Schat and Calnek [31] isolated the MDV-1 strain CVI988 and the MDV-2 strain SB1 from clinically healthy non-vaccinated chickens and were denoted MDV-1 and MDV-2 vaccines, respectively [31]. Unlike the convenient usage of the “dry” MDV-3 vaccine, these vaccine strains needed to be cell-associated in order to be effective. Further to the limited protective ability of the MDV-2 vaccines against the newer more virulent MDV isolates, they were combined for use with the MDV-3 vaccine. The bivalent vaccine showed an outstanding synergistic protection against vvMDV strains [32,33]. Further, it was revealed that the MDV-1 vaccine, CVI988 (denoted also as Rispens CVI 988 strain, by the name of its developer) was the most efficacious vaccine until today [34]. Moreover, the CVI988 vaccine was able to protect against very virulent+ MDV strains and is still considered the “gold standard” of MDV vaccines.

Although MDV vaccines have been successful in protecting chickens against tumors and mortality, the MDV vaccines do not provide sterilizing immunity, and vaccinated chickens still can support replication and shedding of virulent viruses [3,15]. The widespread use of MDV vaccines worldwide is thought to have contributed to the evolution towards greater virulence. Reddy et al. [35] reviewed the four strategies that are being employed by researchers in their effort to generate improved MDV vaccines: (a) serial passages in vitro in cell cultures to attenuate the virulent MDV; (b) serial back-passages of fully attenuated avirulent MDVs in chickens to improve in vivo replication and protection; (c) insertional mutagenesis of viral promoter/enhancer sequences; (d) development of next-generation vaccines based on viral gene functions. Prevention continues to be a challenge in spite of the massive research on MDV.

## 4. The Oncogene Carried by MDV

The innovative discovery of the MDV *meq* gene by Dr. H.-J. Kung and collaborators in 1992 [36] established the notion that this gene is the major gene responsible for the MDV oncogenicity, present in virulent strains and in CVI988 and in strains of lower virulence. The *meq* gene is an oncogene, encoding a leucine-zipper DNA-binding protein vvMDV and in vv+MDV pathotypes. The *meq* protein appears in two configurations, a 339 amino acid unspliced open reading frame and a larger form of *meq* of 398 amino acids, resulting from an in-frame insertion of 180 bp and having multiple duplications of a C-terminal proline rich repeat [37]. The *meq* leucine-zipper protein has a trans-activation N-terminal basic leucine-zipper domain and a C-terminal proline-rich trans-repression domain [38,39,40]. The *meq* gene activities are mediated by its dimerization with itself, as well as with c-Jun like proteins such as JunB, c-Jun, and c-Fos. The *meq* gene C-terminus binds also to cellular transcription factors that are involved in cell division and cycle regulation, such as SNF, ATF, CREB and HB-EGF [39]. The *meq* protein interacts also with proteins without a leucine zipper domain, such as the cellular tumor suppressors p53, retinoblastoma gene, the cyclin-dependent kinase 2, and the heat shock protein Hsp70 [41,42,43]. The *meq* N-terminal portion contains a “PLDLS” amino acid motif that interacts with CtBP co-repressor terminal binding protein-1, forming a meq-CtBP dimer [36,41,42,43,44]. 

The strongest association with the observed increased virulence is the polymorphisms identified at the C-terminal domain of the *meq* encoded oncoprotein, the *meq* encoded major oncoprotein, and transcription factor. Despite the rather low evolutionary rate of double-stranded DNA viruses [45,46,47,48], it has been reported that the *meq* gene is evolving at a much faster rate than most genes in double-stranded DNA viruses [49,50], concurrently with the stepwise evolution of MDV virulence [37,50]. Padhi and Parcells [48] and Trimpert et al. [49] confirmed that *meq* gene sequences not only evolved at a much faster rate than most dsDNA viruses, but its evolutionary rate was comparable to the evolutionary rate of RNA viruses, including the influenza virus. By analyzing the *meq* gene sequences of 84 MDV strains, Padhi and Parcells [49] estimated that the MDV mean evolutionary rate was 10 to 1000 times greater than the mean evolutionary rate of other dsDNA viruses, namely, 1.02 × 10^−4^ mutations per nucleotide per cycle of virus replication (mut/nt/rep), as compared to the range of 10^−5^–10^−7^ mut/nt/rep for herpesviruses. Moreover, the *meq* gene evolutionary rate is much higher than other viral genes in MDV.

The great divergence of the *meq* gene sequences enabled Shamblin et al. [37] to characterize distinctive polymorphisms and point mutations that correlated with the virus virulence. The ability to determine neurovirulence of serotype 1 isolates of MDV by their *meq* gene sequence was confirmed by Renz et al. [51]. In addition to the ability of the actual MDV virulence, the *meq* gene sequences enabled the performance of epidemiological studies that proved useful in revealing epidemiological linkages between various field strains in numerous countries, like China [52], India [53], Poland [54] and Egypt [55]. Recent studies included MDV isolates from Israeli poultry that were collected between the years 1990–2019. Additional confirmation for the functional significance of the *meq* gene sequence in commercial flocks was provided in the recent studies on Italian commercial chicken flocks affected with MD [56] and on MDV tumor-bearing turkey [57].

In addition to the epidemiological information, the *meq* gene sequence revealed the geographical restriction and geographically dependent evolution, as demonstrated by Trimpert et al. [49], who exposed the independent emergence of North American and Euroasian viruses.

The nucleotide-based classification of MDV virulence based on their *meq* gene is highly advantageous as compared to the classical gold standard method of pathotype determination [58]. The gold standard method of pathotype determination compared the course of infection of isolates with that of specific MDV virus prototypes on line 15 × 7 chicks. As this pathotype classification assay was difficult and not feasible for use worldwide, Dudnikova et al. [59] developed an alternative “best fit” pathotyping assay. Although the “best fit” pathotyping assay was simplified, it still employed a long-term experimental infection trial in MDV vaccinated and unvaccinated one-day old SPF chicks that were challenged with virulent isolates at 5 days of age, and then were housed for an additional 56 days in isolator units for observation. The molecular virulence determination is much more rapid and can be performed in standard molecular sequencing laboratories. The pathotype determination of field isolates demonstrated that the number of the four-proline stretches (PPPP) in the *meq* gene transactivation domain are an indicative marker for the pathogenicity of MDV strains isolated from chickens; the most virulent isolates showed the lowest number of PPPP repeats, unlike the attenuated and the low pathogenicity isolates, which showed a highest number of repeats, suggesting that the *meq* gene configuration is indicating the degree of GaHV-2 attenuation [38,52,57]. Conradie et al. [60] provided a deeper insight into the oncogenicity of MDV by showing that the 180-bp insertion in the transactivation domain of the long version of the *meq* gene is greatly influencing the viral telomerase RNA expression via the cellular *myc* gene.

Recently Brown et al. [61] reported for the first time that the *meq* protein can physically, and potentially functionally, interact with the chicken anemia virus (CAV) apoptin protein transactivating the CAV transcription [62]. CAV is an economically important immunosuppressive virus of chickens that causes anemia, lymphoid atrophy, morbidity, and mortality and synergizes with many co-infecting pathogens, including MDV, as demonstrated for avian respiratory viruses [22]. The molecular basis of MDV-CAV interactions is still obscure, and it is unknown. The study implied that the MDV *meq* protein interacts and inhibits the CAV apoptin protein, probably promoting cellular transformation and tumor formation.

## 5. The Functional Significance of the Conformational Epitopes on the MDV Immunodominant Protein

The biologically active membrane glycoproteins are recognized among the herpesviruses as the immunogenic proteins, which are important for the virus antigenicity, cell-attachment, virus-cell membrane fusion and other specific activities. The antigenic epitopes of the immunodominant antigens of MDV were characterized by immunoblotting using convalescent chicken sera from MDV-affected and tumor-bearing commercial birds and include the glycoproteins A (gA) and B (gB) [63]. Glycoprotein B is homologous and conserved in many herpesviruses [64,65,66]. Genomic analyses as well as molecular and antigenic properties indicate that MDV gB is the homolog of HSV-1 gB [67]. MDV gB has been described as a complex of three glycoproteins: gp100, gp60, and gp49. Further, by immunoblotting MDV-infected cell extracts under conditions of minimal denaturation (i.e., without heating or reduction by 2-mercaptoethanol before the SDS-PAGE separation) it was revealed that MDV gB is composed of two high molecular mass oligomers of ≥300 and 230 kDa in unheated cell extracts [63,67]. Upon heating the MDV, gB forms a heat-stable disulfide-linked dimer of 200 kDa consisting of 130 kDa monomers [63,66].

The similarity between MDV and HSV-1 gBs in their high molecular mass oligomeric conformation and the conservation of gB functions among the various herpesviruses prompted us to explore their antigenic relationship. Indeed, the MDV and HSV-1 gBs share two-ways cross-activities [67,68]. We demonstrated for the first time that the MDV gB heat-labile oligomers possess conformational epitopes shared with the human HSV-1 gB heat-labile oligomers. That finding further indicates the common role of gB among herpesviruses. By immunoblotting the cell lysates of MDV and HSV-1-infected cell cultures of denatured, and native cell lysates from MDV and HSV-1 infected cell cultures with convalescent chicken anti-MDV strain RB1B, and human anti-HSV1 gB sera we showed the cross-reactivity, as both MDV and HSV-1 gBs were detected [67].

More advanced studies unveiled the biological activity of MDV gB in its heat-stable and heat-labile configurations in inducing the production of virus-neutralizing antibodies. The virus neutralizing activity was demonstrated using monospecific antibodies to the heated and to the unheated oligomeric MDV and HVT gB [68,69]. The monospecific antibodies were obtained from convalescent chicken sera by immunoaffinity purification on nitrocellulose strips on which the heat-stable dimer or monomer [69] or the heat-labile 230 kDa MDV gB oligomer were blotted [70]. The heat-stable MDV gB components presented strain and serotype-restricted neutralizing epitopes as the specific neutralization index of reactivity was the highest to chicken embryo fibroblast cells infected with a serotype 1 MDV isolate [68]. In addition, the monospecific antibodies neutralized MDVs of the 3 serotypes in a serotype-specific manner, as the highest activities of the 3 monospecific antibodies, the anti-200 kDa, anti-130 kDa, and anti-60 kDa of serotype 1 convalescent serum reacted with MDV serotype 1-infected cells with a higher specific activity than with cell cultures infected with MDVs of serotypes 2 and 3. The monospecific antibodies to the 230 kDa heat-labile oligomers of HVT (serotype 3) and of MDV-B (serotype 1) neutralized the MDV-infected cells with the 3 serotypes in a serotype-restricted manner, indicating that these oligomers presented discontinuous neutralizing epitopes [68]. By these studies we showed that the MDV gB contains both type-specific and type-common antigenic domains, and that the epitopes that elicit neutralizing antibodies are both continuous and discontinuous epitopes. Pereira et al. [71] showed the presence of both type-specific and type-common domains on HSV-1 gC (glycoprotein C) using monoclonal antibodies, however, the dissection of the chicken MDV convalescent serum in terms of various antibodies with various neutralizing activities is distinctly significant.

The demonstration that the MDV gB exists as heat-labile oligomers in its native form resembles the presence of herpes simplex virus 1 (HSV1) gB as a native conformational entity on the infected cell membrane. Membranal HSV gB was described to consist of heat-labile multiple oligomeric species in the 200–300 kDa range [72] or as dimers (200 kDa) of 110 kDa monomers [73]. The functions of these oligomers in viral infectivity included virion to cellular membrane fusion and formation of the virion envelope and spikes [74,75,76].

In contrast to the vast body of studies on the biological properties of discontinuous conformational epitopes of human herpesviruses, the investigation of continuous and discontinuous antigenic epitopes important for viral infection and protection of veterinary herpesviruses is still limited. Our studies conveyed the only description of MDV native epitopes of the immunodominant proteins. In that respect, at present continuous and discontinuous epitopes were identified only on the bovine herpesvirus type 1 gB [77]. Similar to our findings, the HSV-1 gB was described to oligomerize through an essential intermolecular interaction of a 28-amino acid domain and a non-essential upstream that site [78]. The demonstration of the labile formation of the biologically active MDV gB discontinuous epitopes was also consistent with the low pH dependency of the conformation and oligomeric state of gB [79] and its activity in viral fusion to cell membrane and to its entry into the infected cells [80].

In retroviruses, a similar situation regarding the state-of the art of the contribution of 3D antigenic epitopes in virus biology was described only for the reticuloendotheliosis virus [81], in contrast to human retroviruses, where numerous studies were published [82], describing immunodominant proteins possessing both continuous and discontinuous epitopes.

## 6. Molecular Recombination Involving MDV

Multiple viral infections of chickens involving MDV may enable genetic exchanges between the co-infecting viruses. The events involving MDV and an additional DNA virus include three studies in chickens. The first study describes molecular recombination between the fowlpox virus (FPV) [83] and MDV [84]. Although the rate of DNA recombination is supposed to be even lower than events which involve recombination in RNA viruses, Brunovskis and Velicer [84] provided evidence that several FPV genes have homologs in the MDV genome. The second study that disclosed a concrete relevance of in vivo molecular recombination events between DNA viruses was provided by the recombination events that occurred between the live infectious laryngotracheitis virus (ILTV) vaccines that circulated simultaneously in Australia, the Australian A20 and SA2 ILTV vaccines and the European Serva ILTV vaccine [85]. By Simplot analysis two points of crossover corresponding to recombination regions were identified in the genome alignments. As a result of the molecular recombination, two virulent recombinant viruses were associated with outbreaks causing mortality rates of up to 17.6%. The third study employed the next generation sequencing that facilitated that the demonstration of molecular recombination events are quite often in human and in veterinary alphaherpesviruses [86]. Recombination events between virulent and vaccine MDV strains were demonstrated and implicated in impacting viral evolution by altering selective pressures and dissemination of virulent strains.

MDV is involved in molecular interactions between DNA and RNA viruses. Integration of retroviral sequences into the herpesvirus genome was documented in vitro by co-infecting CEF cultures with MDV and the retroviruses reticuloendotheliosis virus (REV) and avian leucosis virus (ALV) [87,88,89,90,91], and reviewed by Kawaguchi and Mikami [92], Bronovskis and Kung [93] and Kung [94]. By co-cultivating MDV and REV in the same tissue culture dish Jones et al. [91] created the first recombinant virus, RM1, which was characterized both molecularly and biologically as having an altered in vitro replication and in vivo biological properties [95]. The RM1 was named using the initials of its two progenitor viruses, REV and MDV. However, co-cultivation of MDV with one of the retroviruses is not the only mechanism by which retroviruses recombine with MDV. Sakaguchi et al. [96] and Endoh et al. [97] reported retroviral LTR integrations into MDV not as a result of co-cultivation of both viruses, but instead, as a result of culture maintenance in host cells that contained avian endogenous viruses. The retrovirus recombination process with MDV occurs because retroviruses integrate into any double stranded (ds) DNA for replication. The LTRs, were predominantly integrated into clusters gathered at the junctions between the unique (long or short) and the terminal or internal repeated MDV fragments (TRL and TRS and IRL and IRS) [93].

Having experienced the relatively efficient creation of recombinant viruses in vitro, we questioned whether retroviruses also integrate into DNA viruses in vivo, in birds, during multiple viral infections. If such process would occur, serious consequences might follow; recombinant MDV might possess altered biological properties, and relatively known features associated with infection by these viruses may be altered resulting in unknown and unpredictable patterns of disease. Putative features, where changes might be of biological significance such as, pathogenicity, virus spread, antigenicity and immunogenicity. Changes in the latter two may lead to changes in the ability of specific vaccines to protect against diseases. We analyzed commercial birds that had acquired naturally mixed infections, chickens infected experimentally with prototype strains of MDV and ALV-J and commercial chickens infected experimentally with virus obtained from cases of double infection with MDV and ALV-J in the same commercial flock or the same bird from that flock [97]. The main findings indicated that integration events happened at various rates depending on the experimental system used. In commercial flocks, molecular recombination events occurred in about 2.5% of 2926 DNA samples [98]. Increasing virus adaptation to laboratory conditions appeared to increase the rate of retrovirus LTR integration into MDV. For the first time MDV co-infections with each of the three avian retroviruses, REV, ALV, and ALV-J, resulted in retroviral LTR integration into MDV in vivo as well as in vitro [98], leading to the creation of multiple types of chimeric quasispecies in dually-infected birds. Since the DNA was extracted from many cells, it was not possible to say whether this represented a true quasispecies in a single cell or unique integrations that occurred in separate cells. Zhang et al. [99] demonstrated the spontaneous recombination of MDV and retroviruses in Chinese commercial flocks. These viruses were disseminated among commercial chicken flocks where enhanced pathogenicity was observed. The extrapolation of in vitro and in vivo system is not straightforward because there are confounding variables, such as the influence of immune responses of the host and different target cell types.

In summary, we highlighted several aspects of the MDV biology, with the awareness that the ground is much wider, and that in each publication different angles are enlightening. Specifically, the recent development of the novel approaches to monitor the vaccine application efficacy using feathers and the use of the *meq* gene diversity for the determination of the MDV virulence and molecular epidemiology is reviewed. For the first time, we review the relatively unknown studies showing the importance of the conformational epitopes of the immunodominant MDV gB in virus neutralization, as well as their serotype- and strain-specific activity, that is a topic that did not receive much attention regarding avian herpesviruses, in general, and particularly regarding GaHV-2. In addition, the molecular recombination between DNA-DNA and DNA-RNA viruses was also highlighted.
